# Boys demonstrate greater knee frontal moments than girls during the impact phase of cutting maneuvers, despite age-related increases in girls

**DOI:** 10.1007/s00167-023-07340-z

**Published:** 2023-02-22

**Authors:** Mohammadhossein Ghasemi, Haraldur Björn Sigurðsson, Þórarinn Sveinsson, Kristín Briem

**Affiliations:** 1 Department of Physical Therapy, University of Iceland, Reykjavík, Iceland; 2 Research Centre of Movement Science, University of Iceland, Reykjavík, Iceland

**Keywords:** Anterior cruciate ligament (ACL), Biomechanics, Risk-factor, Maturity, Sports tasks

## Abstract

**Purpose:**

Anterior cruciate ligament (ACL) injury rate is low among children, but increases during adolescence, especially in girls. Increases in the knee valgus moment within 70 ms of contact with the ground (KFM_0-70_) may explain the sex-specific increase in the risk of ACL injury. The purpose of the study was to investigate sex-dependent changes in the KFM_0-70_ from pre-adolescence to adolescence during a cutting maneuver (CM).

**Methods:**

Kinematic and kinetic data during the CM task, performed before and after physical exertion, were recorded using a motion capture system and a force plate. A total of 293 team handball and soccer players, aged 9–12 years, were recruited. A number of those who continued sports participation (*n* = 103) returned five years later to repeat the test procedure. Three mixed-model analysis of variance (ANOVA) for repeated measures tests were used to determine the effects of sex and age period on the KFM_0-70_ (1: with no adjustment, 2: adjusted for repeated measurements, and 3: additionally adjusted with hip and knee joint frontal plane kinematics).

**Results:**

Boys had significantly higher KFM_0-70_ than girls at both age periods (*p* < 0.01 for all models). Girls, not boys, demonstrated significantly increased KFM_0-70_ from pre-adolescence to adolescence. Importantly, this was fully explained by kinematic variables.

**Conclusion:**

Although the marked increase in KFM_0-70_ seen in girls may play a role in their risk of ACL rupture, the higher values demonstrated by boys during CM reflect the complexity of multifactorial biomechanical risk factor analysis. The role of kinematics in mediating the KFM_0-70_ provides means for modification of this risk factor, but as boys had higher joint moments, continued investigation into sex-dependent biomechanical risk factors is warranted.

**Level of evidence:**

II.

**Supplementary Information:**

The online version contains supplementary material available at 10.1007/s00167-023-07340-z.

## Introduction

An ACL injury is one of the more serious knee injuries sustained by athletes participating in sports involving jumping and cutting tasks, with an injury incidence for individuals in their twenties reported as 154 per 100,000 [26]. The likelihood of concomitant injury to other knee structures is very high and the most younger individuals undergo ACL reconstruction surgery. This has serious consequences including high treatment costs, long-term absence of the athlete from training and competition, a decrease in the athlete’s performance, further joint injuries (e.g., osteoarthritis) [34] and greater risk of a second ACL injury, especially for young individuals [35]. Most ACL injuries occur in a non-contact mechanism [8] where there is a rapid change in speed and direction of movement (e.g., when landing from a jump and during cutting movements), which is commonly performed in multidirectional sports, such as team handball and soccer.

In recent decades, efforts have been made to identify risk factors and mechanisms of ACL injury to potentially screen and find individuals who are more prone to this injury. An increased rate of ACL ruptures is seen during adolescence, in particular for female athletes [26]. Changes that happen to female athletes during that time [1, 7, 12, 25, 27, 28, 36] are therefore targets for injury preventative measures and risk factor studies. Biomechanical [3, 9, 12] and neuromuscular [12, 18] factors have been the focus of research studies since that line of research is most likely to yield modifiable targets for preventative studies. Cross-sectional studies have shown sex-dependent kinematic and kinetic differences during cutting maneuvers (CM) [27] and drop jump tasks [4] in children. However, previous prospective studies conducted for age period differences were limited due to a short follow-up time (1–3 years) [12, 15, 28] or insufficient sample size [25].

Another difficulty with identifying changes in biomechanics that may lead to ACL injury is knowing what to look at. Recent studies have shed some light on the particulars of the injury mechanism such as how quickly (around 50 ms) after ground contact the injury occurs [19, 20]. The multi-planar nature of the injury has been demonstrated in a cadaver study [3] and combinations of trunk and lower limb kinematics during injury have been described [16]. The frontal plane knee moment (KFM) is a strong candidate risk factor although studies have shown mixed results [14, 22, 24]. Hewett et al. (2005) showed that some female athletes who demonstrated high peak knee valgus moment later went on to injure their ACL [14]. In contrast, Krosshaug et al. (2016) and Leppänen et al. (2017) observed that the peak knee valgus moment was not associated with the risk of ACL injury during vertical drop jump task [22, 24]. However, none of these studies extracted the peak joint moment within the specific timeframe relevant for ACL injury (immediately after foot–ground contact) or during the CM. Unlike the drop jump, a CM requires more contributions from the frontal plane. If the KFM during the ACL injury timeframe explains the increased injury risk observed for adolescent female athletes, it should be expected to increase markedly during maturation when performing movements with frontal plane requirements. Hence, the present study aimed to prospectively investigate if there are differences between female and male athletes in how the KFM changes during the transition from youth to adolescence. It was hypothesized that girls would display higher KFM than boys, and that these differences would become more pronounced after the transition from pre-adolescence to adolescence.

## Materials and methods

The study was approved by the Icelandic National Bioethics Committee VSNb20112020011/03.7). This is a secondary analysis of a completed longitudinal laboratory study. Children involved in team sports (handball and soccer; *n* = 293), were recruited at age 9–12 years old. Five years later, 177 of them now aged 14–17 years old returned to participate in the test procedure again. Those who had not continued in their sport from pre-adolescence to adolescence (*n* = 74) were removed. Thus, the data of 103 participants were used for further analysis (demographic characteristics in Table 1).

**Table 1:** Demographic characteristics of participants by sex and age period

Subjects	Number	Age period	Age (year)	Height (cm)	Weight (kg)	Dominant leg
Overall	103	Pre-adolescent	10.8 ± 0.8*^13^	150.3 ± 8.0	40.2 ± 8.0	86 right, 4 left*^13^
		Adolescent	15.8 ± 1.1	173.1 ± 9.0	65.7 ± 11.1	96 right, 7 left
Boys	39	Pre-adolescent	10.6 ± 0.8*^6^	150.6 ± 9.1	40.1 ± 7.9	30 right, 3 left*^6^
		Adolescent	15.7 ± 1.1	181.1 ± 8.7	71.2 ± 13.7	34 right, 5 left
Girls	64	Pre-adolescent	10.8 ± 0.8*^7^	150.1 ± 7.4	40.3 ± 8.1	56 right, 1 left*^7^
		Adolescent	15.9 ± 1.0	168.3 ± 4.9	62.3 ± 7.6	62 right, 2 left

The sign * indicates the number of subjects with missing data

### Eligibility criteria

The inclusion criteria were active participation in team sports (soccer, team handball, or both) at the pre-adolescent age and in one of the five local participating clubs. The exclusion criteria were history of lower limb ligament or muscle rupture or any orthopedic problems precluding them from active participation. The subjects and their guardians provided written informed consent before commencing the test procedure during both visits.

### Task procedure

After measuring height, weight, and distance from iliac crest to the lateral malleolus, athletes warmed up on a stationary bicycle for five minutes at a self-selected pace. A set of 46 retro-reflective markers were used to define and track body segments (trunk, pelvis, thighs, shanks, and feet) [4]. After a static measurement, 12 anatomical markers (malleoli, femoral condyles, trochanters, iliac crests) were removed to allow free movement of the subject. Data were collected with an eight-camera motion capture system (Qualisys Oqus, Gothenborg, Sweden) sampling at 200 Hz (pre-adolescent athletes) or 400 Hz (adolescents) and two force plates embedded in the floor (AMTI, Watertown, USA) sampling at 2000 Hz.

After 1–3 familiarization trials for each task, subjects performed five CM tasks against a dummy opponent for each leg [27] before and after completing a five minute physical exertion task [4]. The order of movement tasks was randomized. For the CM task procedure, participants stood in a ready-position and on a verbal signal performed a quick antero-lateral step in a self-selected cutting angle (range 10–170°) using their preferred technique. The distance of the athlete to the force plate was adjusted such that their preferred technique would have them land on the force plate.

### Data processing

Visual3D software (C-Motion, USA, version 6) was used to construct a model and calculate joint kinematics and kinetics. The resultant signals were low-pass filtered at 20 Hz. The time of ground contact was defined as the first frame with a vertical ground reaction force greater than 5 N. Kinematic variables were extracted at the time of initial contact, including frontal plane hip angle (HFA_IC_) and sagittal and frontal plane knee angles (KSA_IC_ and KFA_IC,_ respectively). Also, the KFM was extracted as the peak value within the first 70 ms after ground contact and normalized by body mass (referred to as KFM_0-70_ for the remainder of this text). Time to reach peak KFM_0-70_ (TKFM_0-70_) was also calculated. The normalized trunk–foot distance at initial contact (NTFD_IC_) has been suggested as a potentially important variables in the ACL injury mechanism [30] and was therefore calculated at initial contact as the medio-lateral distance between the foot and trunk segment center of mass using Formula 1:

1 $${\text{NTFD}}_{{{\text{IC}}}} = \frac{{\sqrt {\left( {{\text{X}}_{{{\text{trunk}}}} - {\text{X}}_{{{\text{foot}}}} } \right)^{2} + \left( {{\text{Y}}_{{{\text{trunk}}}} - {\text{Y}}_{{{\text{foot}}}} } \right)^{2} } }}{{\text{Total height}}}$$

### Statistical analysis

First the outcome variable (KFM_0-70_) was transformed (ln[1 + y]) to correct for skewness and variance heterogeneity. A mixed-model analysis of variance (ANOVA) was used to analyze the transformed KFM_0-70_ values. For all models, an initial full factorial model was used, where sex (girl and boy), age period (pre-adolescent and adolescent), leg (left and right), and exertion (before and after physical exertion) were factors, and the identification number was a random coefficient (random intercept) to adjust for repeated measurements (Model 1). Then random coefficients (random slopes) for age period, leg, and exertion factors were included to adjust for the variability in the effects of these factors on the outcome (Model 2). Models were compared using the Bayesian Information Criterion (BIC) index for mixed models to confirm that the random effects improved the model fit. Lastly, other biomechanical variables related to ACL injury (KSA_IC_, KFA_IC_, HFA_IC_, TKFM_0-70_, and NTFD_IC_) were added as covariates to evaluate the extent to which observable movement patterns influence the KFM_0-70 _ (Model 3). Different combinations of these covariates were tested and the model that had the lowest BIC value was used. Non-significant 3- and 4-way interactions were removed from the models as they did not improve the model fit. All statistical tests were conducted with mixed models module (GAMLj version 1.5.0) [13] for Jamovi software (version 2.3.13) at the significance level of 0.05. Satterthwaite’s method was used to calculate *p* values. The sample size calculation for the first phase of this study (*n* = 293) did not include outcome variables that were used in the current (phase 2) secondary analysis. An attempt was made to recruit all participants who were still involved with team sports and 103 participants returned. Power calculation revealed that this sample size (39 boys and 64 girls) has statistical power to detect effect size (Cohens d) of 0.37 (alpha = 0.05, beta = 0.90).

## Results

### The demographic characteristics of subjects

The demographic characteristics for boys, girls, and overall groups in pre-adolescent and adolescent age periods are seen in Table 1.

### The comparison of different mixed-model tests for transformed KFM_0-70_ values

Random components (slopes) for age period, leg, and exertion all yielded lower BIC values of the mixed-model fit (Supplementary file; Table I) and were included for Model 2 (Table 2). The lowest value of the BIC in mixed-model analysis was found when applying the KFA_IC_ and HFA_IC_ as covariates (Supplementary file; Table II). Based on these findings, age period, leg, and exertion were used as random coefficients and the KFA_IC_ and HFA_IC_ as covariates for Model 3 (Table 2).

**Table 2:** The comparison of p values, fitting indices and random components, for the transformed KFM_0-70_ outcome variable between different mixed models

Variables	Model 1: No adjustment	Model 2: three random slopes	Model 3: three random slopes, two covariates*
	*P* values	*P* values	*P* values
**Factors**			
Sex	< 0.001	0.002	< 0.001
Leg	< 0.001	< 0.001	0.008
Exertion	n.s	n.s	n.s
Age period	n.s	n.s	< 0.001
Sex ✻ Leg	< 0.001*	0.021*	n.s
Sex ✻ Exertion	n.s	n.s	n.s
Leg ✻ Exertion	n.s	n.s	n.s
Sex ✻ Age period	< 0.001*	0.040*	n.s
Leg ✻ Age period	0.002*	0.047*	n.s
Exertion ✻ Age period	0.006*	0.003*	< 0.001*
Sex ✻ Age period ✻Exertion			0.001*
**Model fit**			
R-squared Conditional	0.303	0.523	0.617
R-squared Marginal	0.039	0.042	0.218
BIC	− 6031	− 7706	− 8817
**Random components**			
Subject (intercept) variance	0.009	0.007	0.007
Leg variance		0.011	0.009
Exertion variance		0.002	0.001
Age period variance		0.027	0.022
Residual variance	0.023	0.016	0.014

The sign * indicates significant *P*-value for interactions (*P* < 0.05)

Two selected covariates were KFA_IC_ and HFA_IC_

### The comparison of transformed KFM_0-70_ values among factors

**Fig. 1:**
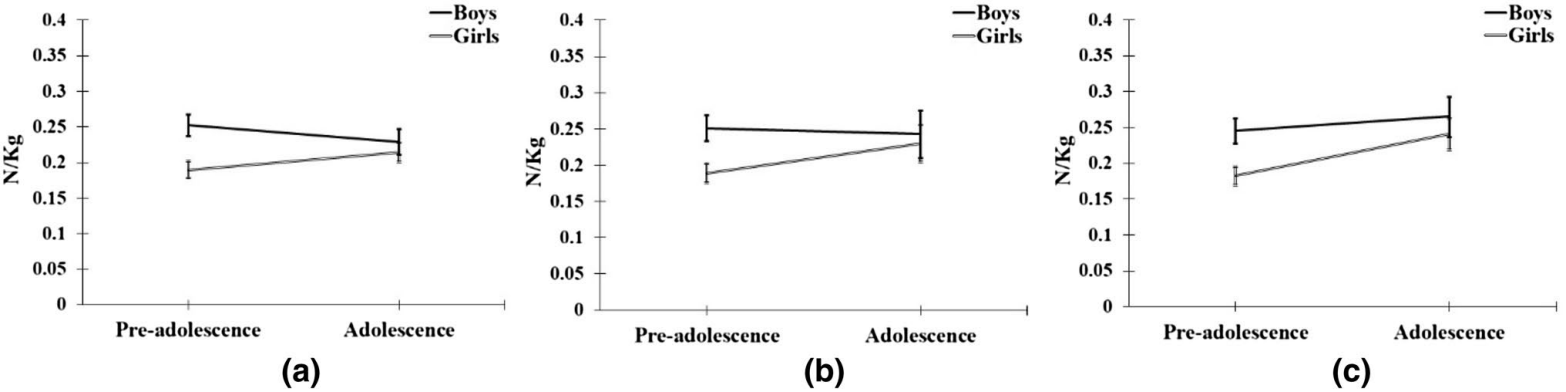
Interactions between sex and age period for the KFM_0-70_ outcome variable in three different mixed models: no adjustment (**a**), three random slopes (**b**), and three random slopes plus two covariates (**c**). Back-transformed adjusted means and 95% confidence intervals are shown

There was a main effect of sex and leg in all three models and a main effect of age period in model 3 (Table 2). There were two-way interactions for sex*age period in Models 1 and 2 but not Model 3 (Table 2). Models 1 and 2 demonstrated an increase in transformed KFM_0-70_ values from pre-adolescence to adolescence for girls (from 0.19 ± 0.01 to 0.21 ± 0.01 and from 0.19 ± 0.01 to 0.23 ± 0.02 Nm/kg, respectively), while boys demonstrated a decrease from pre-adolescence to adolescence age period (from 0.25 ± 0.01 to 0.23 ± 0.02 and from 0.25 ± 0.02 to 0.24 ± 0.03 Nm/kg, respectively) (Fig. 1). However, when adjusting the mixed-model with random slopes and covariates (Model 3), both girls and boys showed an increase in transformed KFM_0-70_ values from pre-adolescence to adolescence (from 0.18 ± 0.01 to 0.25 ± 0.02 and from 0.25 ± 0.02 to 0.27 ± 0.03 N/kg, respectively) (Fig. 1).

**Fig. 2:**
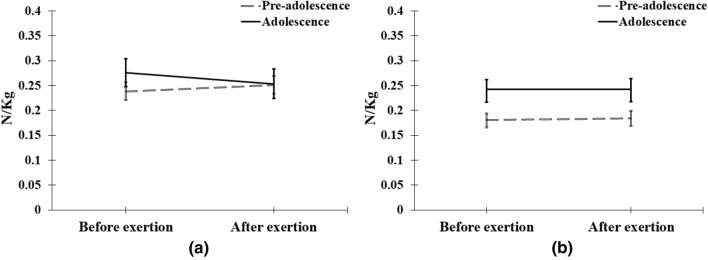
The effect of exertion in boys (**a**) and girls (**b**) in pre-adolescence and adolescence age periods. Back-transformed adjusted means are shown and 95% confidence intervals

One significant 3-way interaction was found for KFM_0-70_ and that was in the adjusted Model 3 for sex*age period*exertion (*P* < 0.05) (Table 2). The interaction was due to a post-exertion decrease in KFM_0-70_ seen in adolescent boys but not girls (Fig. 2).

## Discussion

The most important finding of the present study was that boys had a greater KFM than girls during the 0–70 ms timeframe during which an ACL rupture would occur. While an increase over time was seen for females, this increase explained less than 4% of the variance and disappeared in statistical model that adjusted for kinematics. The results are congruent with previous studies that have identified longitudinal changes in the KFM for female athletes [12, 15]. Unlike those studies, the focus of this analysis was on the timeframe immediately following initial ground contact as well as a CM task to make the results more relevant for the ACL injury mechanism [19, 20]. This may, in part, explain the fact that female subjects in the current study did not have higher KFM values compared to the males at either time point and so the increase observed in the girls served only to close the gap between the sexes. During a drop jump, a movement pattern of dynamic valgus which is commonly observed for female athletes [12] may contribute to the much greater KFM differences reported previously for such a task [15]. During CM, there are some additional potential confounders which may differ between the sexes and explain the higher values for boys, such as cutting angle and movement speed [21]. In terms of injury risk, girls may have a lower threshold for tolerating sudden KFM loads as their ACL is smaller and not as stiff [6]. Girls are also more likely to experience concurrent forces after initial ground contact which may, in part, influence ACL injury risk [32].

Previous studies have hypothesized that the ability of female adolescents and adults to attenuate forces contributing to the KFM is decreased due to sex-dependent anatomical [17], muscular [1], neuromuscular [1, 23, 25, 28], and biomechanical [10, 23, 28] differences. In the present study, when the model was adjusted for hip and knee frontal angle, no differences in the KFM_0-70_ were detected between the sexes. The two frontal plane covariates (KFA_IC_ and HFA_IC_) are considered biomechanical risk factors for ACL injuries [2, 11, 14]. One interpretation of this result is that sex-based differences in kinematics are driving the increase of KFM observed for female athletes. The models without kinematics had an explained variance of ~ 4% for the fixed effects of Model 1 (R-squared Marginal; Table 2), while the model with kinematics had an explained variance of ~ 20% for Model 3 (R-squared Marginal; Table 2). The sex and age group therefore only explain a fairly insignificant percentage of the KFM_0-70_ compared to the kinematics. The random effect structure explained a further 40% of the variance (R-squared Marginal vs. R-squared Conditional for Model 3, Table 2), indicating the possibility that there are variables not captured in the fixed effects which can be influential on the KFM_0-70_. Another paper based on the same adolescent girls as the current study reported that the KFM waveform was highly variable in this timeframe and that patterns with high KFM values were relatively rare [31]. The closer the peak values occur to the initial contact, the greater the influence of the impact with the ground must be and less the influence of joint angles. In the current study, the distance between foot and trunk did not influence the KFM value, but was associated with greater odds of showing the early peak waveform in the previous study [31].

There was a three-way interaction between sex, age group, and exertion because boys responded to the physical exertion with a lower KFM_0-70_. While the original intent of the physical exertion protocol was to produce fatigue [4], the effects in the adolescent age group might simply be a warm-up effect rather than fatigue. However, these differences were small and unlikely to be of clinical significance.

The strengths of this study include its prospective nature and a sample size allowing for the statistical approach taken. The primary limitation of the current study is the secondary, exploratory, design which makes it more prone to generate non-reproducible findings. Physical maturity and physical fitness are probable confounders when assessing the different changes with maturation, and these were not assessed in the study. The direction of this bias would be toward greater KFM for boys, as was observed in the study.

There are known limitations regarding the precision of optical marker-based motion capture systems such as those used in the current study [5]. While knee kinetics during cutting maneuvers is known to the fairly reliable [29], errors in the estimate of KFM may be as high as 0.375 Nm/kg. The results in the manuscript are reported with two decimals of precision, but it is not known whether the methods are precise enough to determine the last decimal. The calculation of the KFM depends on the frontal plane angle of the knee, which is known to have a systemic bias with increasing flexion angles [33]. Small errors in the placement of knee markers can also shift the center of rotation and artificially create a correlation between flexion and abduction. However, given the large sample size of the current study and the random uniform distribution of such errors, this effect is unlikely to affect the results.

These findings are clinically relevant for the potential attenuation of ACL injury risk. Targeting the frontal plane kinematics at the moment of impact has the potential to reduce the KFM of the impact phase of CMs. While girls showed more increases with age, the results indicate that interventions could be aimed at all athletes who show impact phase kinematics consistent with higher KFM. Despite girls having a higher risk of ACL injury, boys in the study had an overall higher KFM. Clinicians should be aware that addressing the KFM alone might not be sufficient to reduce the risk of ACL injuries.

## Conclusion

Girls demonstrated an increased KFM_0-70_ during adolescence but to a smaller extent than previous studies have indicated. The increase seems driven by the knee and hip frontal plane kinematics, which still are unlikely to fully explain sex-dependent differences in risk of ACL injury. The clinical implications are that frontal plane kinematics should be targeted when attempting to influence the KFM_0-70_ while considering other biomechanical risk factors.

## Supplementary Information

Below is the link to the electronic supplementary material.

Supplementary file1 (DOCX 58 KB)
