# Supplementary material for: Boys demonstrate greater knee frontal moments than girls during the impact phase of cutting maneuvers, despite age-related increases in girls
Source: Knee Surg Sports Traumatol Arthrosc. 2023 Feb 22;31(5):1833–9. doi: 10.1007/s00167-023-07340-z (PMC10090008; doi:10.1007/s00167-023-07340-z)
Supplement: Supplementary file 1 — Supplementary file1 (DOCX 58 KB) [file 167_2023_7340_MOESM1_ESM.docx]

| **Table I** The effect of random coefficient on mixed model outputs for transformed KFM_Ph_ values | | | |
| --- | --- | --- | --- |
| **Random Coefficient** | **BIC** | **R^2^ Marginal** | **R^2^ Conditional** |
| **Age, Leg, Exertion** | **-7638** | **0.042** | **0.524** |
| Age, Leg | -7633 | 0.043 | 0.511 |
| Sex, Age, Leg, Exertion | -7594 | 0.042 | 0.524 |
| Sex, Age, Leg | -7597 | 0.043 | 0.511 |
| Age, Exertion | -7020 | 0.044 | 0.436 |
| Age | -7025 | 0.044 | 0.426 |
| Sex, Age | -6999 | 0.044 | 0.426 |
| Sex, Age, Exertion | -6984 | 0.044 | 0.436 |
| Leg | -6404 | 0.038 | 0.383 |
| Leg, Exertion | -6391 | 0.038 | 0.390 |
| Sex, Leg | -6378 | 0.038 | 0.383 |
| Sex, Leg, Exertion | -6356 | 0.038 | 0.390 |
| **Only identification number** | **-5961** | **0.040** | **0.303** |
| Exertion | -5949 | 0.040 | 0.308 |
| Sex | -5943 | 0.040 | 0.303 |
| Sex, Exertion | -5923 | 0.040 | 0.308 |
| Important values were bolded.  Factors were “Sex”, “Age”, “Leg”, and “Exertion”.  No “Covariates” were set for the model.  The identification number was set as a “Random Coefficient” for all models.  The order of row is based on the *BIC* values from the highest to lowest. | | | |

| **Table II** The Effect of covariates on mixed model outputs for transported KFM_Ph_ values | | | |
| --- | --- | --- | --- |
| **Covariates** | **BIC** | **R^2^ Marginal** | **R^2^ Conditional** |
| **KFA_IC_, HFA_IC_** | **-8817** | **0.218** | **0.617** |
| KFA_IC_, T KFM_Ph_, HFA_IC_ | -8744 | 0.218 | 0.618 |
| KSA_IC_, KFA_IC_, HFA_IC_ | -8736 | 0.219 | 0.617 |
| KSA_IC_, KFA_IC_, T KFM_Ph_, HFA_IC_ | -8720 | 0.218 | 0.618 |
| KFA_IC_, HFA_IC_, NTFD_IC_ | -8337 | 0.259 | 0.611 |
| KSA_IC_, KFA_IC_, HFA_IC_, NTFD_IC_ | -8323 | 0.26 | 0.611 |
| KFA_IC_, T KFM_Ph_, HFA_IC_, NTFD_IC_ | -8320 | 0.259 | 0.612 |
| KSA_IC_, KFA_IC_, T KFM_Ph_, HFA_IC_, NTFD_IC_ | -8306 | 0.259 | 0.611 |
| KFA_IC_ | -8094 | 0.122 | 0.577 |
| HFA_IC_ | -8084 | 0.116 | 0.549 |
| KFA_IC_, T KFM_Ph_ | -8081 | 0.122 | 0.581 |
| KSA_IC_, HFA_IC_ | -8079 | 0.119 | 0.553 |
| KSA_IC_, KFA_IC_ | -8070 | 0.122 | 0.576 |
| T KFM_Ph_, HFA_IC_ | -8067 | 0.115 | 0.55 |
| KSA_IC_, T KFM_Ph_, HFA_IC_ | -8063 | 0.119 | 0.555 |
| KSA_IC_, KFA_IC_, T KFM_Ph_ | -8057 | 0.122 | 0.581 |
| KFA_IC_, NTFD_IC_ | -7988 | 0.192 | 0.58 |
| KSA_IC_, KFA_IC_, NTFD_IC_ | -7987 | 0.195 | 0.581 |
| KFA_IC_, T KFM_Ph_, NTFD_IC_ | -7971 | 0.192 | 0.581 |
| KSA_IC_, KFA_IC_, T KFM_Ph_, NTFD_IC_ | -7970 | 0.194 | 0.582 |
| HFA_IC_, NTFD_IC_ | -7782 | 0.166 | 0.552 |
| T KFM_Ph_, HFA_IC_, NTFD_IC_ | -7764 | 0.166 | 0.552 |
| KSA_IC_, HFA_IC_, NTFD_IC_ | -7759 | 0.166 | 0.553 |
| KSA_IC_, T KFM_Ph_, HFA_IC_, NTFD_IC_ | -7741 | 0.166 | 0.553 |
| **No covariate** | -7638 | 0.042 | 0.524 |
| KSA_IC_ | -7625 | 0.044 | 0.526 |
| T KFM_Ph_ | -7624 | 0.043 | 0.528 |
| KSA_IC_, T KFM_Ph_ | -7613 | 0.043 | 0.531 |
| NTFD_IC_ | -7589 | 0.122 | 0.536 |
| T KFM_Ph_, NTFD_IC_ | -7572 | 0.121 | 0.537 |
| KSA_IC_, NTFD_IC_ | -7565 | 0.122 | 0.537 |
| KSA_IC_, T KFM_Ph_, NTFD_IC_ | -7548 | 0.121 | 0.537 |
| Important values were bolded.  Factors were “Sex”, “Age”, “Leg”, and “Exertion”.  “Age”, “Leg”, “Exertion”, and “Identification number” were set as the “Random Coefficients”.  The order of row is based on the *BIC* values from the highest to lowest. | | | |
